# Singular Cause of Death

**Published:** 1848-08

**Authors:** 


*Singular cause of death !*—The municipal regulations of the city of New York, require that the physician attending every case of disease, which proves fatal, shall deliver, in writing, a statement of the cause of death, for registration. The following has been handed us by a friend, who states that it is a literal transcript of the cause of the death of a patient, as registered, in the language of the medical attendant :—

"*This woman was died, because she did die, and she was die of vickness and she could not live.* DR. VANDERHIDEN.

